# Preparing for pandemics: a systematic review of pandemic influenza clinical management guidelines

**DOI:** 10.1186/s12916-022-02616-6

**Published:** 2022-11-07

**Authors:** Ishmeala Rigby, Melina Michelen, Vincent Cheng, Andrew Dagens, Dania Dahmash, Samuel Lipworth, Eli Harriss, Erhui Cai, Valeria Balan, Alexandra Oti, Reena Joseph, Helen Groves, Peter Hart, Shevin Jacob, Lucille Blumberg, Peter W. Horby, Louise Sigfrid

**Affiliations:** 1 International Severe Acute Respiratory and Emerging Infection Consortium, Pandemic Sciences Institute, University of Oxford, Oxford, OX3 7LG UK; 2 Bristol Medical School, University of Bristol, Bristol, BS8 1TL UK; 3 Oxford University Hospitals NHS Foundation Trust, Oxford, OX3 9DU UK; 4 Nuffield Department of Medicine, University of Oxford, Oxford, OX3 7BN UK; 5 Bodleian Health Care Libraries, University of Oxford, Oxford, OX3 9DU UK; 6 Department of Veterinary Medicine, University of Cambridge, Cambridge, CB2 1TN UK; 7 Imperial University, London, SW7 2BX UK; 8 Wellcome Trust, London, NW1 2BE UK; 9 Liverpool School of Tropical Medicine, Liverpool, L3 5QA UK; 10 National Institute for Communicable Diseases, Johannesburg, South Africa

**Keywords:** Pandemic, Preparedness, Influenza, Clinical management guidelines, AGREE II, Supportive care

## Abstract

**Background:**

The COVID-19 pandemic has highlighted the importance of evidence-based clinical decision-making. Clinical management guidelines (CMGs) may help reduce morbidity and mortality by improving the quality of clinical decisions. This systematic review aims to evaluate the availability, inclusivity, and quality of pandemic influenza CMGs, to identify gaps that can be addressed to strengthen pandemic preparedness in this area.

**Methods:**

Ovid Medline, Ovid Embase, TRIP (Turning Research Into Practice), and Guideline Central were searched systematically from January 2008 to 23rd June 2022, complemented by a grey literature search till 16th June 2022. Pandemic influenza CMGs including supportive care or empirical treatment recommendations were included. Two reviewers independently extracted data from the included studies and assessed their quality using AGREE II (Appraisal of Guidelines for Research & Evaluation). The findings are presented narratively.

**Results:**

Forty-eight CMGs were included. They were produced in high- (42%, 20/48), upper-middle- (40%, 19/48), and lower-middle (8%, 4/48) income countries, or by international organisations (10%, 5/48). Most CMGs (81%, 39/48) were over 5 years old. Guidelines included treatment recommendations for children (75%, 36/48), pregnant women (54%, 26/48), people with immunosuppression (33%, 16/48), and older adults (29%, 14/48). Many CMGs were of low quality (median overall score: 3 out of 7 (range 1–7). All recommended oseltamivir; recommendations for other neuraminidase inhibitors and supportive care were limited and at times contradictory. Only 56% (27/48) and 27% (13/48) addressed oxygen and fluid therapy, respectively.

**Conclusions:**

Our data highlights the limited availability of up-to-date pandemic influenza CMGs globally. Of those identified, many were limited in scope and quality and several lacked recommendations for specific at-risk populations. Recommendations on supportive care, the mainstay of treatment, were limited and heterogeneous. The most recent guideline highlighted that the evidence-base to support antiviral treatment recommendations is still limited. There is an urgent need for trials into treatment and supportive care strategies including for different risk populations. New evidence should be incorporated into globally accessible guidelines, to benefit patient outcomes. A ‘living guideline’ framework is recommended and further research into guideline implementation in different resourced settings, particularly low- and middle-income countries.

**Supplementary Information:**

The online version contains supplementary material available at 10.1186/s12916-022-02616-6.

## What is already known on this topic?

- Clinical management guidelines (CMGs) are evidence-based tools to facilitate clinical decision-making and access to the best available evidence-based care.
- The CMGs provided early in the COVID-19 pandemic were of limited quality and scope.
- Supportive care is the main treatment for many (re-) emerging infections, and early access to evidence-based supportive care can improve patient outcomes.

## What are the new findings?

- There is limited availability of pandemic influenza CMGs globally, most were developed for upper-middle and higher-income settings.
- Many CMGs were of limited quality and were produced more than 5 years ago, many during the H1N1 pandemic and only one had been updated since then.
- There were limited, heterogeneous and at times contradictory recommendations on supportive care, and limited recommendations for different at-risk populations.
- All CMGs recommended oseltamivir, but with variations in recommendations for pregnant women. Recommendations on additional neuraminidase inhibitors were conflicting, reflecting the limited evidence-base to support recommendations.

## What do the new findings imply?

- The data highlight a need to ensure mechanisms for regular updates of pandemic influenza CMGs are considered at the development stage, to ensure publicly available CMGs are up to date, providing the best available evidence-based treatment and supportive care recommendations, for different at-risk populations.
- The limited scope highlights a need for investments into trials to identify effective treatments and supportive care strategies to benefit patient care and outcomes.
- A living guideline framework with mechanisms for integrating new evidence and dissemination, with close links between researchers and guideline developers from different settings, is needed to improve the quality, inclusivity, and availability of evidence-based care recommendations.
- Further research is needed into the implementation of CMGs from development to the front line in different settings and contexts.

## Background

Influenza pandemics are one of the greatest global health threats, potentially causing millions of deaths and huge socioeconomic disruption. The ever-present threat of pandemic influenza and experiences with the COVID-19 pandemic emphasise the critical importance of pandemic preparedness.

The 1918 influenza pandemic, previously labelled “the deadliest pandemic of modern age” [1], gave us an illustration of how fatal an influenza pandemic can be, with an estimated 50–100 million deaths globally [1]. The more recent influenza A (H1N1) 2009 pandemic, despite a lower case-fatality rate than predicted, reminded us of the risk to human health from a novel virus [2]. Most recently, the COVID-19 (SARS-CoV-2) pandemic, with more than six million deaths reported (as of 11th May 2022), highlights our ongoing global vulnerability to emerging viral infections and a need to incorporate lessons learnt to strengthen our preparedness for future outbreaks [3]. The COVID-19 pandemic has been a reminder of how rapidly respiratory infections transmit globally, resulting in morbidity, mortality, economic, societal and health system disruptions [4–7]. It has further demonstrated that we are still not adequately prepared for a pandemic global response, as the Global Preparedness Monitoring Board highlighted in October 2019 [8].

Public health and government preparedness activities have largely focused on surveillance, reporting, epidemiological modelling, and prevention and control [9, 10]. However, preparedness for the optimal clinical management of new and emerging influenza infections is equally important. This includes the need to identify and mitigate poor outcomes in those most at risk by identifying and implementing optimal supportive care strategies and host-directed and antiviral therapies [11]. Limited data on the clinical effectiveness of influenza antivirals [12, 13] and potential resistance [14] to recommended drugs (adamantanes, zanamivir, and oseltamivir) pose additional challenges. Thus, the risk to global health security from the emergence of novel pandemic influenza virus strains, including a drug-resistant strain, remains high [15, 16].

The COVID-19 pandemic has also highlighted issues of inequity in access to care globally [17]. Variation in clinical care between sites may impact patient outcomes and may also confound trial results and impede evaluation of medical countermeasures. This variation was demonstrated during the 2013-2016 Ebola Virus Disease (EVD) outbreak in West Africa, where the mortality of patients receiving care in the United States or Europe was lower (18.5%) than in West Africa (37 to 74%) [18]. The difference in mortality was partially attributed to the lack of adequate supportive care in West Africa [18]. This was also an issue in the PAmoja TuLinde Maisha (PALM) trial, a randomised control trial (RCT) of therapeutics for EVD conducted in West Africa, which was impacted by limited access to standardised supportive care measures [19].

Access to evidence-based clinical management guidelines (CMGs) can be a vital tool in the clinical response to a pandemic [20–22]. Clinical Management Guidelines (CMGs) are recommendations aimed at guiding and standardising clinical decision-making to benefit patient outcomes [23–29]. The decline of in-hospital case-fatality rate for COVID-19 may exemplify how improvements in the clinical management of emerging infections may improve patient outcomes [30, 31]. Although this decline is multifactorial, a change in clinical practice (e.g. better management of severe cases) was a notable factor [30, 31]. The standardisation of evidence-based care may facilitate implementation of multisite interventional studies to identify the best supportive care, treatment, and vaccination strategies. The early stages of emerging pandemics place a burden on CMGs to be responsive despite limited evidence and to be regularly updated and disseminated as new evidence rapidly emerges. Reviews of CMGs for other high-consequence infectious diseases have identified concerning variation in availability and quality of CMGs and in inclusivity of recommendations targeted at different at-risk populations [29, 32, 33].

The aim of this review is to identify gaps in access to evidence-based pandemic influenza CMGs for different at-risk populations globally and assess variations in supportive care and treatment recommendations that may have an impact on outcomes and implementation of clinical trial response to pandemics.

## Methods

We conducted a systematic review of the literature focused on pandemic influenza CMGs. This review followed the Cochrane systematic review guidance [34] and was structured according to the Preferred Reporting Items for Systematic Reviews and Meta-Analyses (PRISMA) statement guidelines [35]. This review is part of a wider project evaluating the availability, inclusivity, scope and quality of clinical management guidelines for high-consequence infectious diseases (HCID), registered with PROSPERO (International prospective register of systematic reviews) (CRD42020167361) [36].

### Search strategy

We searched three databases (Ovid Medline, Ovid Embase, Turning Research Into Practice (TRIP)) and a guideline repository (Guideline Central) from 1 January 2008 to 23rd June 2022. The date was restricted from 2008 onwards to include recent CMGs incorporating recommendations based on the latest developments, whilst also ensuring we included those produced in response to the influenza A pandemic (H1N1, pdm09) [37, 38].

We validated the search strategy by testing the terms before finalising the search strategy. We identified keywords and phrases from an initial set of pandemic influenza guidelines, identified from clinical experts and hand-searches in the planning stages. From these, we identified associated MeSH/Emtree terms, subject headings, and indexes from specific databases. The search strings were then tested against the initial standard set to ensure the quality of the final search strings used for the review.

We complemented the search with a grey literature search which was completed on the 16th of June 2022. We searched Google Scholar to retrieve relevant records from 1 January 2008 with the first 500 hits screened. Additionally, to identify a globally representative sample of international and national CMGs, we conducted a google search using pre-defined keywords in Spanish, French, German, Mandarin, Arabic, and Russian. Finally, we contacted clinical network members of the International Severe Acute Respiratory and Emerging Infection Consortium (ISARIC) [39] in regions where no CMGs were identified via the database and grey literature searches. We specifically searched for CMGs including recommendations for influenza A and several of its variants (H1N1, H5N1, H7N3, H7N7, H7N9 and H9N2). A full search strategy is available in Additional file 1: S1.1-S1.3.

### Eligibility criteria

We defined CMGs as documents (developed using systematic or non-systematic methodologies) that provided recommendations on supportive care or empirical treatments to guide practice, in line with the WHO’s (World Health Organization) definition. These included guidelines aimed at children (0 to <18 years old), adults, pregnant women, older people (> 65 years old), and/or people living with HIV [40]. Supportive care was defined as therapeutic interventions (e.g. fluids/supplemental oxygen/ventilatory support) which aim to optimise the patient’s physiological status and are not directly targeted at the underlying pathogen or pathophysiological process, as per the definition by US CDC (Center for Disease Control and Prevention) [41]. We included CMGs that focused on pandemic influenza defined as a novel influenza A virus of any zoonotic origin with pandemic potential [38]. Pandemic influenza, although more rare than seasonal influenza, has the capacity to infect a large number of people, due to no or limited prior exposure. We included results in any language. Where multiple versions existed, we included only the most recent version. Documents were excluded if they were local standard operating procedures or guidelines only focused on infection prevention and control, animals, diagnostics procedures, non-traditional medicine, or seasonal influenza, without providing any treatment recommendations.

### Screening

After deduplication, search results were screened independently by two reviewers using Rayyan, a systematic review software [42]. The articles were first screened by title and abstract, followed by full-text screening. Any disagreements were resolved by consensus or by a third reviewer. The CMGs published in non-English languages were translated using Google translate for rapid translation of the full document, then screened, data extracted, and critically appraised by a reviewer with good to excellent knowledge of the language.

### Data extraction

We extracted data as per the methodological requirements described in the design and conduct of systematic reviews of clinical guidelines produced by Johnston et al. [43] Data extraction was performed by one reviewer using a standardised data extraction form which we previously validated [32]. Any disagreements were resolved by involving a third reviewer. We extracted data on bibliography, issuing organisation, year issued, region aimed at, inclusivity (populations covered), and scope (supportive care, and empirical treatment recommendations) (Additional file 2: Table S2.1). Data on the methods used to grade and formulate the recommendations was extracted and categorised (e.g. systematic, expert consensus, a combination of methods or based on other guidelines).

### Data analysis

The extracted data was analysed to assess availability, inclusivity scope and quality using descriptive analysis. Availability was assessed by whether up-to-date CMGs could be identified. The CMGs were stratified by origin: (1) international organisations (e.g. WHO) and (2) national organisations (e.g. MoH (Ministry of Health) or National Public health institutes). Inclusivity was assessed on the inclusion of recommendations targeting the whole population, including infants, children, adults, pregnant women, older people, as well as people living with HIV/immunosuppression. Statistical analysis was performed in the R language for statistical computing version 4.0.2 [44, 45] with the ggplot2 library used to produce graphics [46].

### Quality assessment

The quality was assessed by two reviewers independently using the Appraisal of Guidelines for Research & Evaluation (AGREE) II tool [47]. The tool consists of 23 criteria across six domains: (1) scope and purpose, (2) stakeholder involvement, (3) rigour of development, (4) clarity of presentation, (5) applicability, and (6) editorial independence. Each criterion was independently assessed by two reviewers on a seven-point Likert scale, from 1 (strongly disagree) to 7 (strongly agree) as per the AGREE II tool user manual [47]. For CMGs with limited information on their methodology, attempts were made to identify further information on related webpages or by contacting the organisation.

Overall domain scores were calculated as per the AGREE II tool user manual, converting the sum of individual scores from each reviewer into a standardised percentage of the maximum score possible for each domain [47]. Guidelines were considered of high quality if they scored more than 60% in domain three (rigour of development; as this is considered a high-quality indicator) [48], and two other non-specified domains. If a CMG scored more than 60% in any three or more domains, not including domain three, it was considered to be moderate quality. If they did not reach any of these criteria, a CMG was assessed as being low quality [47]. Additionally, each CMG was also given an overall quality assessment score which was informed by the domain scores, ranging from one to seven (high-quality score ≥6; medium-quality score 4–5; low-quality score ≤ 3), together with a recommendation for use with or without further modifications. The CMGs with a total overall quality score of 1 were not recommended for use. Those with a total overall scores of 2-5 were recommended for use with modifications and those that scored 6–7 recommended for use without modifications.

### Patient public involvement

There was no patient public involvement in this project due to the ongoing pandemic constraints.

## Results

Of a total of 1817 records identified, 48 met the eligibility criteria (Additional file 3: Fig. S3.1) [49–96]. No additional guidelines were identified through the clinical networks that had not been already included.

### Characteristics of included CMGs

Many (65%, 30/48) CMGs focused on clinical management of A(H1N1) [49–65, 67–76, 78, 79, 92], 4% (2/48) on A(H7N9) [80, 93], 2% (1/48) on H5N1 [91], and 29% (15/48) were generic influenza pandemic CMGs [66, 77, 81–90, 94–96]. Fifty-eight per cent (28/48) were produced in 2009–2010 in response to the A(H1N1) influenza pandemic [49–75, 92]. Only 17% (8/48) [85–90, 93, 94, 96] were produced or updated within the last 5 years and none were ‘living CMGs’. Most (90%, 43/48) were produced by a national organisation, 10% (5/48) by an international organisation [67, 71, 78, 92, 94]. The CMGs were produced in Spanish (40%, 19/48) [50, 51, 55–58, 60, 61, 63, 65, 69, 72, 74, 76, 79, 82, 83, 87, 90], English (31%, 15/48) [49, 53, 59, 62, 67, 71, 73, 77, 78, 80, 85, 92, 94–96], Chinese (8%, 4/48) [84, 88, 89, 93], French (4%, 2/48) [68, 70], Italian (4%, 2/48) [52, 66], Japanese (4%, 2/48) [86, 91], German (2%, 1/48) [75], Portuguese (2%, 1/48) [64], Romanian (2%,1/48) [54], and Russian (2%, 1/48) [81]. Twenty-seven percent (13/48) of the CMGs used systematic methods [52, 60, 63, 64, 72, 75, 80, 81, 83, 88, 90, 94, 95], 21% (10/48) expert consensus [49, 51, 55, 57, 67, 69, 77, 78, 84, 92], 13% (6/48) a combination of systematic methods and expert consensus to formulate their recommendations [53, 58, 65, 71, 79, 86]. Eight CMGs were adopted from international CMGs (e.g. from the WHO and US CDC) [51, 54, 63, 81, 84, 88, 89, 96], whereas 38% (18/48) of guidelines did not clearly disclose the methods used to formulate their recommendations.

### Availability

**Fig. 1:**
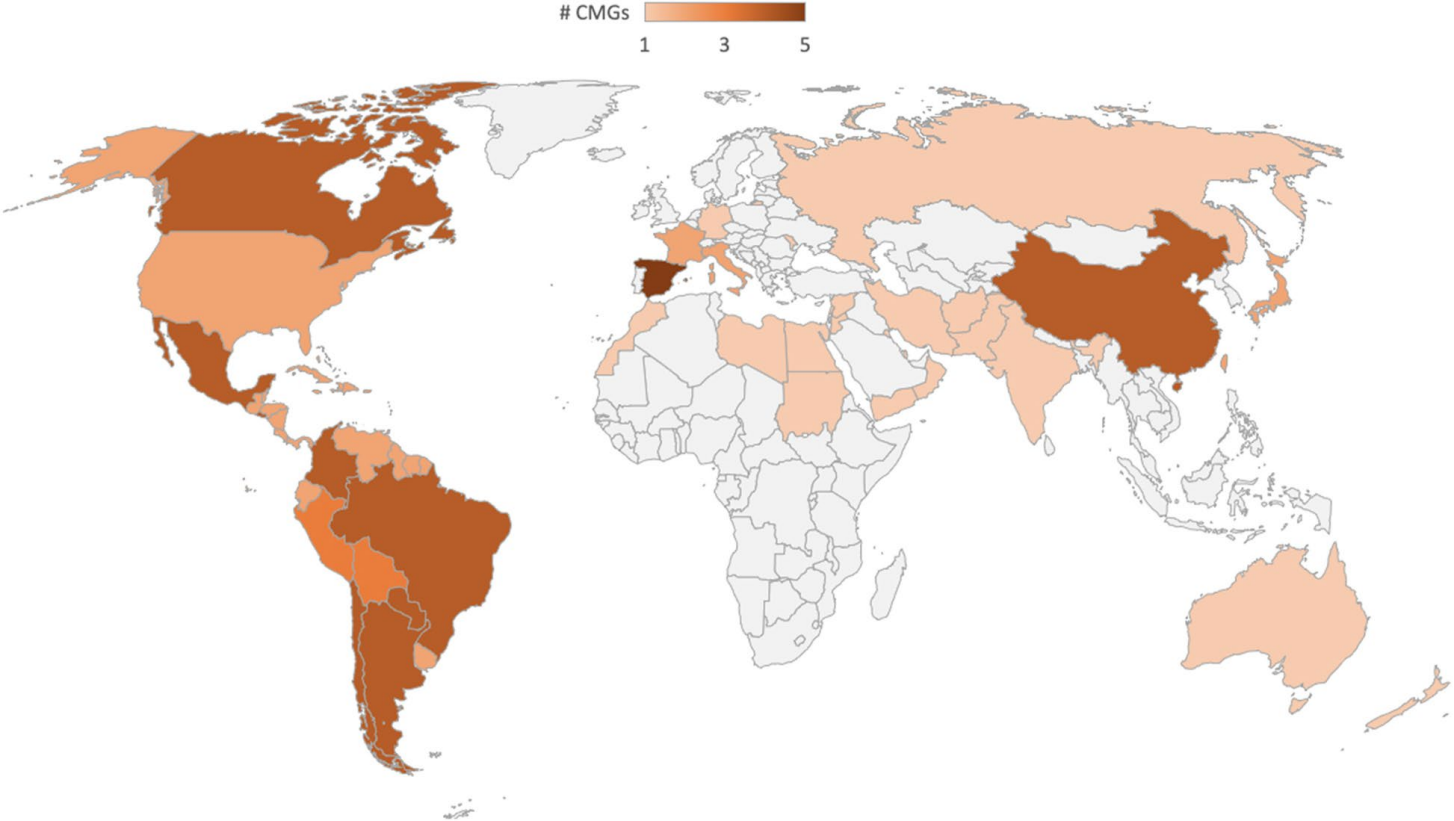
Geographic coverage of the identified CMGs. The shading represents the number of CMGs identified by country. In addition, there were two worldwide CMGs developed by the WHO identified [71, 94]. Abbreviations: CMG, clinical management guideline

Most CMGs were aimed for high- (42%, 20/48) [49, 52, 53, 55–57, 66, 68, 70, 72, 75, 77, 79, 80, 82, 83, 85, 86, 91, 96] and upper-middle- (40%, 19/48) [50, 51, 54, 58–60, 63, 64, 69, 73, 74, 76, 81, 84, 87–89, 93, 95] income countries followed by lower-middle-income countries (8%, 4/48) [61, 62, 65, 90], and 10% (5/48) for a specific region or global use (Table 1, Fig. 1) [67, 71, 78, 92, 94]. No national CMGs were produced in low-income countries (Additional file 4: Table S4.1).

**Table 1:** CMGs by pandemic influenza type, region, and country income classification

	A (H1N1)	A (H7N9)	A (H5N1)	Influenza A	Pandemic influenza	Total
**World Bank region classification**						
East Asia and Pacific	3	1	1	1	4	10
Europe and Central Asia	10	-	-	-	2	12
Latin America and the Caribbean	14	-	-	-	4	18
Middle East and North Africa	1	-	-	-	-	1
North America	-	1	-	-	3	4
South Asia	1	-	-	-	-	1
Global	1	-	-	-	1	2
**Total**						48
**World Bank income classification**						
High-income countries	11	1	1	-	7	20
Upper-middle income countries	12	1	-	1	5	19
Lower-middle income countries	3	-	-	-	1	4
Low-income countries	-	-	-	-	-	-
Global or regional	4	-	-	-	1	5
**Total**						48

The table presents the number of identified CMGs by influenza type, region and World Bank classification [97]

### Inclusivity

Most CMGs (75%, 37/48) included supportive care recommendations tailored for children [49–51, 53–57, 59–67, 69, 71–74, 77, 78, 80–85, 87, 89, 90, 92–95], 54% (24/48) for pregnant women [49, 50, 53, 54, 56–58, 60, 63–66, 68–70, 74, 77, 82–84, 87, 90, 92, 94], 29% (13/48) for older people [50, 60, 63–65, 69, 74, 77, 82, 84, 90, 92, 94], and 33% (16/48) [50, 56, 57, 63, 65, 69, 74, 76, 77, 82, 90, 92, 94] for the treatment of people who are immunosuppressed and/or living with HIV. Only 21% (10/48) included recommendations for all of these different risk groups [63, 65, 69, 74, 76, 77, 82, 90, 92, 94]. The definition of ‘older people’ varied among CMGs, 44% (21/48) included specific recommendations for people aged over 65, 10% (5/48) for over 60 and 2% (1/48) for people over 50 years old, whereas 44% (21/48) did not provide an age range.

### Quality assessment

**Fig. 2:**
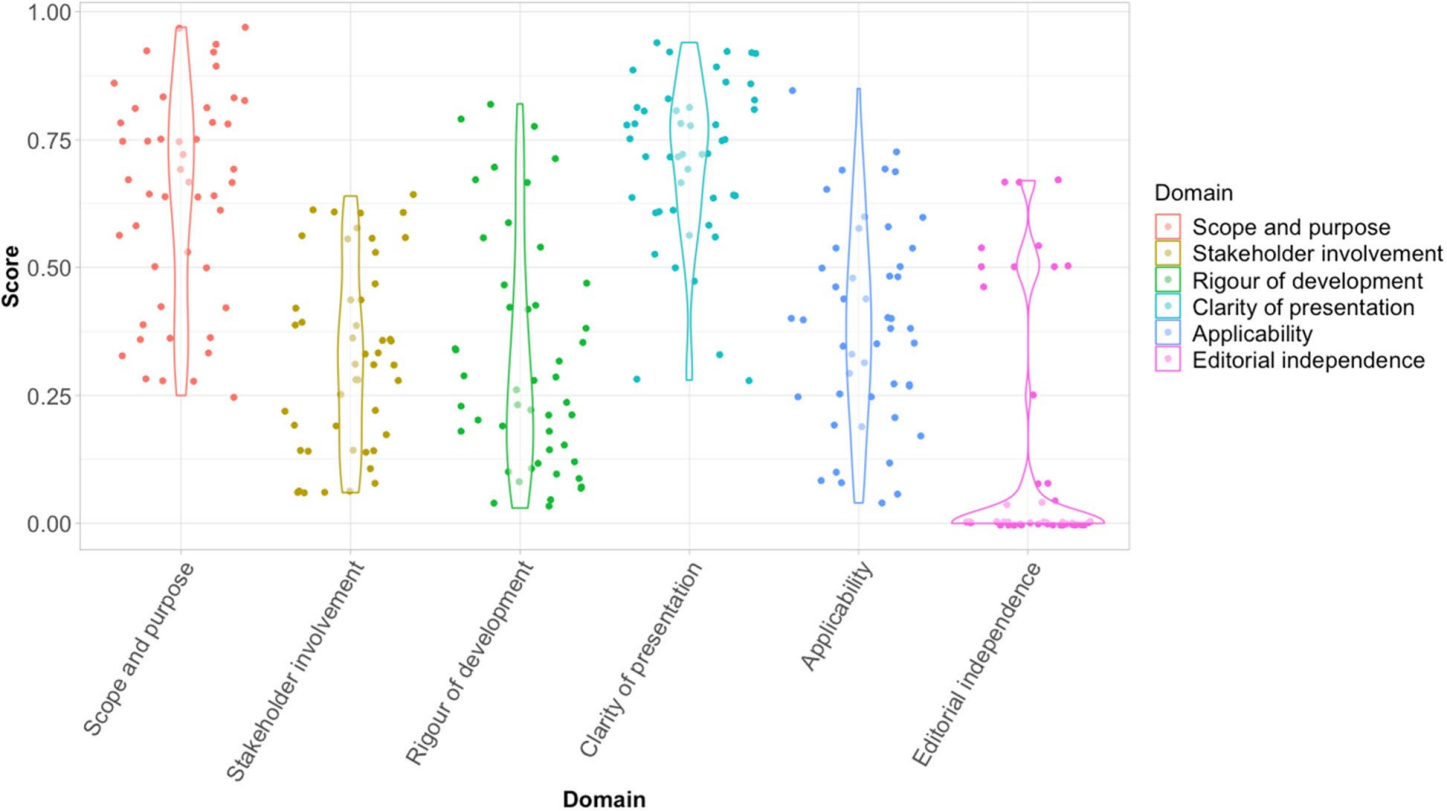
AGREE II domain scores. The violin plots depict the variation in scores of individual CMGs in each domain. Each dot represents a CMG’s proportional score per domain. The width of each curve represents the frequency of CMGs scoring that corresponding value in each domain. Abbreviations: AGREE, Appraisal of Guidelines for Research and Evaluation II; CMG, clinical management guideline

The median overall quality score of all the CMGs was 3 out of 7 (range: 1–7). Most (75%, 36/48) were assessed as of low quality (overall score ≤3) [49, 50, 52–56, 58, 59, 61–66, 68, 73–76, 78, 80–82, 84–93, 95, 96], 15% (7/48) as medium (overall score 4–5) [51, 67, 69–71, 77, 79], and only 10% (5/48) as of high quality (overall score ≥6) [57, 60, 72, 83, 94] (Fig. 2, Table 2). The most recently updated CMG, by WHO, was the most comprehensive guideline, and of the highest quality (overall score 7) [94]. Seventy-seven per cent (37/48) of the CMGs were recommended to be used with further modifications based on the overall AGREE II assessment.

**Table 2:** AGREE II quality assessment

Author/organisation	Focus	Year	Language	D1 (%)	D2 (%)	D3 (%)	D4 (%)	D5 (%)	D6 (%)	Overall score (1: low, 7: high)	Evidence type^a^	GRADE used
Ang, B. et al. [49]	A (H1N1)	2009	English	69	47	54	78	54	0	3	E	N
Arbo, A [76].	A (H1N1)	2009	Spanish	36	28	10	78	38	0	2	U	N
Aristizábal, G. et al. [51]	A (H1N1)	2009	Spanish	94	64	29	86	50	0	4	E	N
Capozzi, C. et al. [52]	A (H1N1)	2009	Italian	75	19	28	72	21	0	3	S	N
Cheng, A. C. et al. [53]	A (H1N1)	2009	English	61	56	42	94	25	54	3	C	N
Cojocaru, V. et al. [54]	A (H1N1)	2009	Romanian	50	53	18	61	35	0	2	U	N
Salud Madrid [55]	A (H1N1)	2009	Spanish	78	33	38	69	27	0	3	E	N
Fernández-Cruz, E. et al. [56]	A (H1N1)	2009	Spanish	39	19	7	61	19	0	2	U	N
GTEI [57]	A (H1N1)	2009	Spanish	75	44	71	81	58	50	6	E	N
GPPI [58]	A (H1N1)	2009	Spanish	69	14	67	58	19	8	3	C	N
Hajjar, L. A. et al. [59]	A (H1N1)	2009	English	64	31	35	72	44	0	3	U	N
Mexico [60]	A (H1N1)	2009	Spanish	89	61	67	81	40	54	6	S	Y
El Salvador [61]	A (H1N1)	2009	Spanish	50	6	9	61	60	0	3	U	N
India [62]	A (H1N1)	2009	English	28	6	11	64	48	0	2	U	N
MOH Argentina [63]	A (H1N1)	2009	Spanish	56	17	21	78	58	0	2	S	N
MOH Brazil [64]	A (H1N1)	2009	Portuguese	81	33	26	86	46	0	3	S	N
MOH El Salvador [65]	A (H1N1)	2009	Spanish	72	22	23	78	48	0	3	C	N
MOH Italy [66]	Pandemic influenza	2009	Italian	36	14	8	47	27	0	1	U	N
PAHO [92]	A (H1N1)	2009	English	64	42	47	83	17	0	3	E	N
PAHO (b) [67]	A (H1N1)	2009	English	78	14	18	89	69	4	4	E	N
Picone O. et al. [68]	A (H1N1)	2009	French	83	28	19	72	40	0	3	U	N
Soria, J. et al. [69]	A (H1N1)	2009	Spanish	67	58	42	72	69	67	5	E	N
France [70]	A (H1N1)	2009	French	75	28	10	75	69	0	4	U	N
WHO [71]	A (H1N1)	2009	English	92	56	34	92	85	0	4	C	N
GT-PBE [72]	A (H1N1)	2010	Spanish	86	44	79	81	29	50	6	S	Y
Lee, PI. et al. [73]	A (H1N1)	2010	English	42	14	15	72	10	0	2	U	N
MOH Mexico [74]	A (H1N1)	2010	Spanish	28	6	4	64	50	0	2	U	N
Schaberg, T. et al. [75]	A (H1N1)	2010	German	33	31	47	72	40	67	3	S	N
Arbo Sosa, A and Araya S [76].	A (H1N1)	2011	Spanish	25	6	5	28	8	0	1	U	N
Fiore, A. E. et al. [77]	Pandemic influenza	2011	English	81	39	59	75	65	4	4	E	N
Hajjar, Al. S. et al. [78]	A (H1N1)	2011	English	64	36	22	78	44	0	3	E	N
Zhong, N. et al. [95]	Pandemic influenza	2011	English	75	36	32	92	38	50	3	S	N
Rodriguez, A. et al. [79]	A (H1N1)	2012	Spanish	97	61	56	81	60	46	5	C	N
Evans, A G. et al. [80]	A (H7N9)	2013	English	78	56	43	75	31	0	3	S	N
MOH Russia [81]	Pandemic influenza	2013	Russian	75	56	70	56	54	0	3	S	N
Saldías, F [82].	Pandemic influenza	2013	Spanish	42	6	23	64	40	0	2	U	N
Garcia, C. et al. [83]	Pandemic influenza	2014	Spanish	92	61	78	89	48	50	6	S	N
Bin, C. et al. [84]	Pandemic influenza	2016	Chinese	83	39	34	92	6	0	3	E	N
US CDC [96]	Pandemic influenza	2016	English	28	25	13	53	4	0	1	U	N
British Columbia [85]	Pandemic influenza	2017	English	53	31	14	64	33	0	2	U	N
China NHFPC [93]	A (H7N9)	2017	Chinese	58	14	7	56	25	25	1	U	N
JAMRD [86]	Pandemic influenza	2017	Japanese	67	36	29	50	35	4	2	C	N
MOH Colombia [87]	Pandemic influenza	2018	Spanish	83	39	20	67	25	8	3	O+	N
Taiwan CDC [88]	Infl. A	2018	Chinese	64	8	24	92	35	0	3	S	N
China NHFPC [89]	Pandemic infl.	2020	Chinese	67	22	13	81	8	0	3	U	N
Fernandez, O. et al. [90]	Pandemic infl.	2020	Spanish	36	36	21	33	27	0	1	S	N
WHO [94]	Pandemic influenza	2022	English	97	61	82	83	73	67	7	S	Y
MOH Japan [91]	A (H5N1)	-	Japanese	33	11	3	28	13	0	1	U	N

The table presents the CMGs included in the review and the individual domain (D) and overall quality score for each. D1: scope and purpose, D2: stakeholder involvement, D3: rigour of development, D4: clarity of presentation, D5: applicability, D6: editorial independence

Key: D: domain, S: systematic methods, E: expert consensus, C: combination of systematic methods and expert consensus, O: other methods used, U: unclear, Y: yes, N: no

Abbreviations: *CDC* Centre for Disease Control and Prevention, *GRADE* The Grading of Recommendation, Assessment, Development and Evaluation, *GPPI* Grupo Promotor de Políticas Informadas, *GTEI* Grupo de Estudio de Infecciones en el Paciente Crítico, *GT-PBE* Grupo de Trabajo de Pediatría Basada en la Evidencia, *HCID* high-consequence infectious disease, *JMARD* Japan Agency for Medical Research and Development, *MoH* Ministry of Health, *NHFPC* China National Health and Family Planning Commission, *PAHO* Pan-American Health Organisation, *US* United States of America, *WHO* World Health Organization

^a^Methods used to search for evidence

There were wide variations in the individual domain scores which assess different aspects of CMG development. Most CMGs scored well in the “scope and purpose” domain (median (IQR): 67% (48–79%)) and “clarity of presentation” domain (median (IQR): 74% (63–81%)), showing recommendations were usually clearly structured and presented. Generally, CMGs scored lower for “rigour of development” (median (IQR) 25% (14–44%)) domain. This domain is considered a strong quality indicator of a CMG, providing up-to-date, evidence-based information [47]. Similarly, there were lower scores for “stakeholder involvement” (median (IQR) 32% (16–45%)), “editorial independence” (median (IQR) 0% (0–8%), and “applicability” (median (IQR) 39% (25–51%)) domains, which may be partially due to a lack of information provided (e.g. on stakeholder engagement including patients, conflict of interest statements, information to support and monitor implementation, and a process for future revisions).

**Fig. 3:**
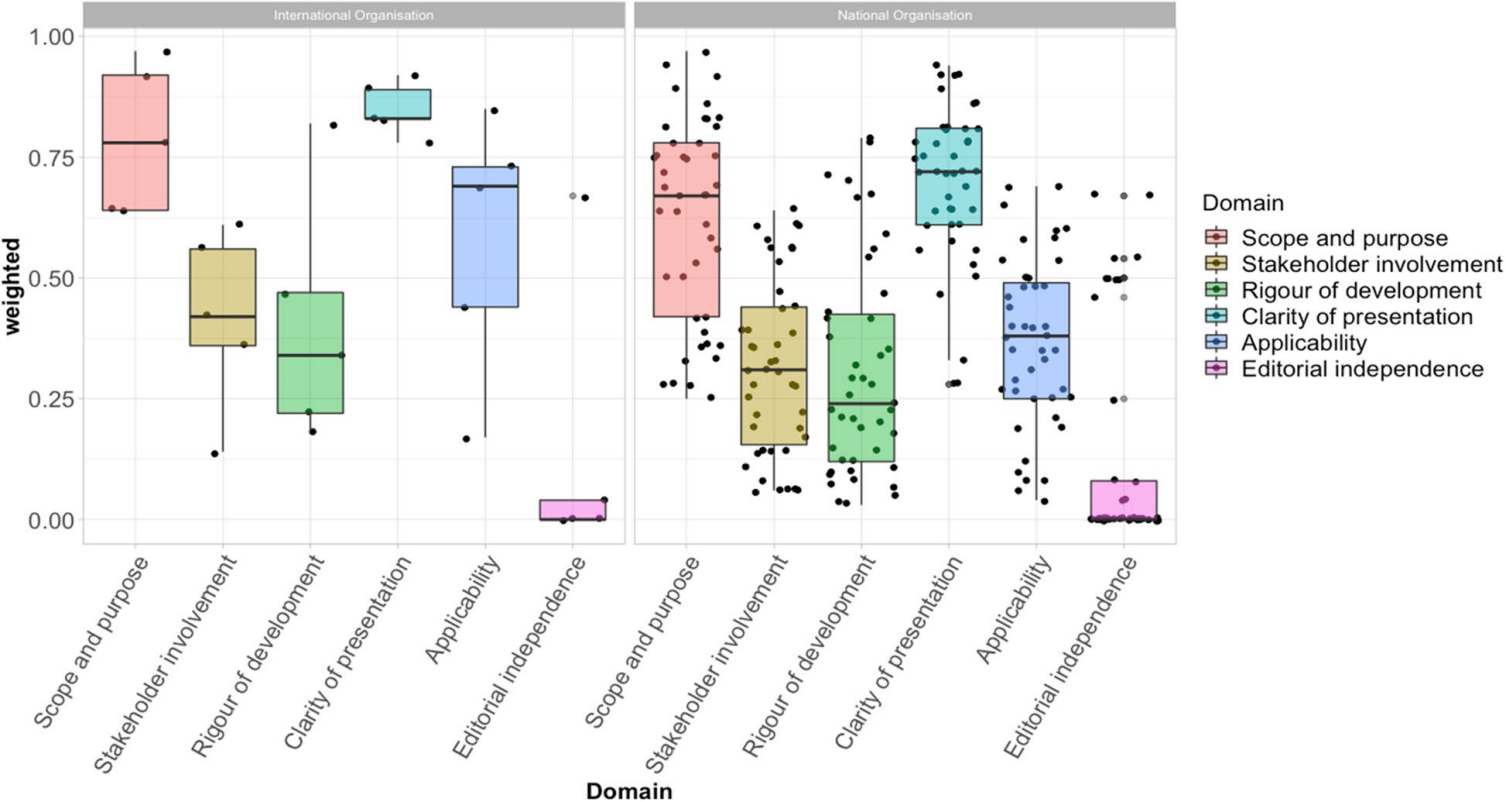
Comparison of the quality between CMGs produced by international and national organisations. The boxplots show the range and distribution of CMG scores by domain, showing CMGs produced by international scored higher, but with a similar pattern, to those produced by national organisations. Domains 1, 2, 3, and 5 showed significant heterogeneity of scores between guidelines, with large interquartile ranges for these domains. Less heterogeneity was observed for domains 4 and 6. Abbreviations: AGREE, Appraisal of Guidelines for Research and Evaluation II; CMG, clinical management guideline

The CMGs produced by international organisations generally scored higher for overall quality (median: 4, IQR: 3–4) compared to those produced by a national organisation (median: 3, IQR: 2–3) (*p*=0.048) (Fig. 3).

### Scope

All CMGs recommended the use or conditional use of Oseltamivir (Additional file 1: Table S4.2). The guideline produced by WHO in 2022, specified to administer oseltamivir as soon as possible in persons with suspected or confirmed influenza virus infection with or at risk of severe illness (i.e. including seasonal, pandemic, and zoonotic influenza) [94]. Further, that this recommendation was based on low-quality evidence for critical outcomes [94].

Other than oseltamivir, 83% (40/48) of CMGs recommended the use or conditional use of zanamivir [49, 50, 52, 53, 55–61, 63–73, 75–84, 86, 88–90, 92, 93, 95, 96], 4% (2/48) amantadine [49, 95], and 4% (2/48) rimantadine [49, 95], whereas the most recent guideline produced by WHO advised against inhaled zanamivir and laninamivir and intravenous peramivir, based on a very low certainty of benefit rather than on evidence of harm [94]. However, they emphasised that this recommendation does not apply if the causative strain is known or at high risk of being resistant to oseltamivir, nor does it apply to intravenous zanamivir [94]. For intravenous peramivir, they cited a lack of evidence on its effectiveness in improving patient outcomes, but that it may be considered in patients unable to take oral or inhaled neuraminidase inhibitors [94]. Advice regarding when to use zanamivir instead of oseltamivir in the other CMGs was brief, 10% (5/48) recommended zanamivir as an alternative when circulating strains were resistant to oseltamivir [49, 53, 56, 60, 64]. One recommended the use of zanamivir alone, or oseltamivir plus an adamantane when the circulating influenza strain is unknown [49]. Furthermore, 56% (27/48) [52, 55, 57, 59, 61, 66, 68, 72, 73, 75–84, 88–91, 93–96] highlighted that antiviral resistance to amantadine and rimantadine should be considered when treating influenza A (H1N1, pdm09) since the risk of resistance is becoming widespread.

There was little variability in the recommended dosage of neuraminidase inhibitors (oseltamivir and zanamivir) for adults and children (including infants). In adults, the recommended dose of oseltamivir, when available, was consistent at 75 mg twice per day for 5 days (once per day for 10 days for chemoprophylactic use) [49–54, 56, 59, 60, 62–64, 66–70, 75, 77–80, 83–85, 87–93, 95], whereas one CMG recommended 15 days [74]. The oseltamivir recommendations for children and infants were also consistent, with recommended dosage based on age and weight (Additional file 4: Table S4.3) [49–54, 56, 59, 60, 62–64, 66–71, 75, 77–80, 83–85, 87–93, 95]. The recommendation for zanamivir was 10 mg inhalation twice per day for 5 days [49, 52, 53, 59, 60, 65–68, 70, 75–78, 82–84, 88–90, 92, 93, 95], or once per day for 10 days for chemoprophylactic use for both adults and children [49, 53, 59, 60, 66, 68, 70, 75–78, 80, 82, 83, 88]. Dosage for other drugs were also recommended but without further indications, such as peramivir (adults: 300–600mg daily, neonates: 6mg/kg, infants: 8mg/kg, and children: 10mg/kg; intravenous, 1–5 days) [84, 86, 88, 89, 93] and umifenovir (200mg, 3 times per day, 5–10 days) [89, 93].

There was generally a consensus in recommendations for pregnant women, 50% (24/48) of CMGs providing advice recommended oseltamivir or zanamivir [49, 51, 53, 54, 56–58, 60, 63–65, 68, 70, 72, 74–77, 82, 83, 87, 88, 90, 96], with some emphasising that pregnant women should only be given the adult dose of oseltamivir if the benefits outweigh the risks [60, 63, 64]. One CMG specifically recommended oseltamivir instead of zanamivir for pregnant women with asthma [58].

#### Corticosteroids

Sixty-three per cent (30/48) [49–53, 55–58, 60, 61, 63–70, 72, 74–77, 82, 83, 85, 87, 90, 96] of CMGs did not provide any guidance on the use of corticosteroids, 15% (7/48) [54, 81, 84, 86, 89, 91, 93] provided limited guidance, simply suggesting that corticosteroids can be considered but provided no further details. Furthermore, 23% (11/48) advised against the use of corticosteroids [59, 62, 71, 73, 78–80, 88, 92, 94, 95], of these, 72% (8/11) [59, 62, 71, 78, 88, 92, 94, 95] advised that a low dose of corticosteroids can be considered for patients in septic shock. The WHO CMG based their recommendations against use of corticosteroids for influenza on observational studies and noted a lack of RCTs [94]. One CMG provided a list of corticosteroids (e.g. dexamethasone and prednisolone) without indicating when they should be used, the dose, or the duration [81]. In one CMG, corticosteroids (a moderate dose for 2 weeks) were recommended in the early stages of respiratory distress syndrome; however, recommended against corticosteroids in the late stages [54]. Of the CMGs that recommended low-dose corticosteroids for patients in septic shock, three [59, 62, 88] further specified that hydrocortisone should be used, with one specifying 50 mg, IV, four times per day [59]. One CMG recommended that low-dose corticosteroids be considered for patients with septic shock who require vasopressors, but did not recommend high-dose systematic corticosteroids outside of clinical trials [71].

#### Antibiotics

Around half of the CMGs (54%, 26/48) recommended antibiotics if a patient shows clinical signs of bacterial pneumonia or secondary bacterial infection [50, 51, 54, 56, 59–62, 65, 68, 69, 71–74, 77–79, 81, 87–89, 91–93, 96]. Forty-two per cent (20/48) [49, 52, 55, 57, 58, 63, 64, 66, 67, 70, 75, 76, 80, 82–86, 90, 95] did not provide any guidance on antibiotics usage, whereas, 4% (2/48) [53, 94] advised against the routine use of antibiotics for influenza-like illness. The recent CMG by WHO recommended not to administer macrolides for the treatment of influenza (citing very low quality of evidence) [94]. The CMGs with empirical antibiotic recommendations advised that they should be directed at bacterial pathogens commonly associated with influenza such as *Streptococcus pneumoniae*, *Staphylococcus aureus*, and *Haemophilus influenzae* [50, 73, 74].

#### Supportive care recommendations

There were limited and varied recommendations on supportive care (Additional file 1: Table S4.2). Most commonly**,** oxygen therapy was addressed in 56% (27/48) of CMGs [50–52, 54, 59, 61, 62, 65, 67–71, 73–75, 79–81, 86, 87, 89, 91, 92, 95, 96]. Oxygen therapy guidance was frequently brief, indicating supplemental oxygen as required to correct hypoxaemia based on clinical condition (severity and oxygen saturation monitoring by pulse oximetry), to maintain a level of pulse oxygen saturation (SpO2) above 90%. For pregnant women, three CMGs specified that oxygen saturation level should be maintained at 92–95% [65, 71, 95]. Only 27% (13/48) of CMGs briefly addressed fluid therapy recommendations [50–52, 54, 59, 61, 62, 65, 67, 82, 87, 92, 95], mainly recommending to maintain proper fluid and electrolyte balance to prevent dehydration for hospitalised and at-home care, without further guidance. Furthermore, 54% (26/48) provided details on at-home care, including analgesic and antipyretics (acetaminophen, paracetamol) for the management of fever or pain and appropriate hydration [50, 51, 55–57, 60–62, 64, 65, 67, 69, 70, 72, 74, 76, 78, 81–83, 85, 87, 89, 92, 95, 96].

## Discussion

Our review highlights the limited global availability of high-quality, up-to-date pandemic influenza CMGs. Although there were a few high-quality CMGs, these were generally produced in high-income or upper-middle-income settings. There were limited CMGs identified from lower-resourced settings which are particularly vulnerable to influenza outbreaks, due to limited healthcare systems and high burden of co-existing diseases such as HIV [98]. During the COVID-19 pandemic, we witnessed how limited infrastructure and lack of access to new technologies and resources, was a barrier for implementation of supportive care, particularly in lower-resourced settings [99]. Additionally, how any setting and healthcare system can become lower resourced during a pandemic [99]. There is a lack of CMGs providing supportive care and treatment recommendations for different at-risk populations such as infants, children, pregnant women, older people and people living with HIV, populations often at higher risk of more severe illness and complications [100]. This may indicate inequity in inclusion of these populations in treatment trials [101]. This is in line with findings from systematic reviews of SARS, MERS and COVID-19 CMGs [33].

Although there was a general consensus in the CMGs on antiviral treatment recommendations for adults and children, there were limited and heterogeneous host-directed, supportive care recommendations provided. The corticosteroid recommendations varied, whilst many CMGs did not provide any guidance on corticosteroids; others, including the most recently developed CMG, advised against administering it [59, 62, 71, 73, 78–80, 88, 92, 94, 95], with a few recommending a low-dose corticosteroid to patients with septic shock [59, 62, 71, 78, 88, 92, 95] or early respiratory distress syndrome [54, 71, 88, 89]. Although there are studies indicating that corticosteroid use may increase mortality and ICU length of stay in patients with influenza [102–104].

Determining the role of oseltamivir is an urgent unmet research need, especially given its cost and widespread use, and adverse reactions [105]. The rapid emergence of antiviral resistance [106] further emphasises the need for a more diverse range of treatments. Whilst there are some anti-influenza therapeutics currently undergoing clinical trials, there are few treatments licensed for use globally [107]. The limited, varied and at times contradictory guidance available illustrates an urgent need for clinical trials to identify optimal treatment strategies, inclusive of the whole population.

Similarly, a review of early pandemic COVID-19 CMGs found inconsistencies in treatment recommendations among CMGs, whereas in some recommended experimental treatments (e.g. hydroxychloroquine), others specified that these should only be used as part of clinical trials [32, 108]. Clinical trials are key for identifying if treatments are effective. Non-evidence-based recommendations and heterogenous treatment recommendations may not only be ineffective, but potentially harmful to patients, and in addition a barrier to the implementation of trials. Further consideration, especially in lower-resourced settings is the utility cost of recommending ineffective treatments. A survey on the implementation of COVID-19 CMGs early during the pandemic identified limited access to supportive care, such as oxygen, especially in low-income countries [99]. For emerging infections where the evidence base may be limited, effective supportive care can improve survival rates, therefore, it is important that CMGs providing evidence-based supportive care recommendations for whole populations are accessible and implementation-supported [18]. Ensuring that CMGs are up to date is crucial to sustain their evidence-base, validity, and credibility; yet most CMGs were produced in response to the H1N1 pandemic (2009) and only one had been updated more recently [94]. Guideline development frameworks recommend regular reviews and CMG updates, every three to 5 years [109]. For emerging infectious diseases, such as influenza and COVID-19 where the epidemiology and new evidence may change rapidly, guidelines need to be flexible and adaptive [33]. Moreover, it is important to not overlook the quality of the CMGs. The low-quality scores in some CMGs may be due to the lack of or limited information presented. Yet, high-quality guidelines contain rigorous methodologies which guideline developers should acknowledge and adopt to facilitate the production of thoroughly produced evidence-based guidelines.

Developing evidence-based CMGs is resource intensive, requiring wide stakeholder engagement, and evidence appraisals, and resources for regular reviews and updates. The low quality of many of the guidelines indicates that this may be beyond the resources available in many nations. The guidelines produced by international organisations that can be adapted and adopted globally may provide a more feasible, robust, and sustainable model. To achieve this, guidelines must be tailored for different regions’ endemicity, risk factors, and drug resistance. Global coordination will reduce the risk of proliferation of heterogenous CMGs with limited scope and value and save valuable resources.

Our study is not without limitations. Although substantial efforts were made to identify CMGs, including targeted searches in different languages, there is still a possibility that some local CMGs were not retrieved. This may partly explain the limited CMGs from low-income countries and the WHO African region especially. Some of the included guidelines were of limited scope, however, after much discussion involving clinicians and global collaborators, these guidelines were included, as they reflect the limited guidance available to clinicians. Despite identifying diverse CMGs in multiple languages, due to translations some nuances of the CMGs may have been lost. Nonetheless, using a diverse team and a combination of search methods, a wide range of CMGs were identified which highlighted concerning gaps in the availability, inclusivity, scope, and quality of available CMGs. Additionally, although the AGREE II tool assesses the methodological quality of CMGs, it does not assess the validity of the treatment recommendations. Despite these limitations, our review identified concerning gaps in the availability and standardisation of pandemic influenza CMGs and limited treatment and supportive care recommendations. The recently updated CMG by WHO addresses some of these limitations, but also highlights that the evidence base is still lacking [94]. Clinical management guidelines are key tools for guiding clinical decision-making, and standardising care to optimise patient outcomes. The COVID-19 pandemic has illustrated the need for rapid clinical management guidance, even when the evidence is scarce. Close collaboration between CMG developers and wider stakeholders such as clinical trial networks, and healthcare professionals should be considered as part of guideline development frameworks for the rapid identification of new evidence and to identify clinical questions in need of an update.

## Conclusions

Our data highlights the limited availability of high-quality, up-to-date pandemic influenza CMGs globally, especially in LMICs. Most of those identified were of limited quality, scope, and inclusivity. The most recent guideline updated this year shows that the evidence-base to support antiviral treatment recommendations is still limited. Our data highlights a need for updating of existing pandemic influenza guidelines, to ensure they provide the latest evidence-based recommendations, inclusive of different at-risk populations. There is a clear role for an improved framework for CMG development, including mechanisms for regular review updates, and dissemination to improve access to evidence-based care recommendations for different at-risk populations. A ‘living guideline’ framework is recommended.

Our data shows an urgent need for trials into effective supportive care, host-directed and antiviral treatment strategies and for new evidence to be incorporated into globally accessible guidelines, to benefit patient outcomes. Moreover, research into the implementation of CMGs in lower-resourced settings.

## Supplementary Information

**Additional file 1: **Details of the search strategy. **S1.1.** Database search strategy. **S1.1.2.** Updated database search strategy. **S1.2.** Google scholar search strategy. **S1.2.2.** Updated google scholar search strategy. **S1.3.** Google engine search strategy.

**Additional file 2: **Data extraction form. **S2.1.** Data extraction form.

**Additional file 3: Additional figure Figure S3.1.** PRISMA diagram

**Additional file 4: Additional tables Table S4.1.** Characteristics of identified CMGs. **Table S4.2.** CMG recommendations for treatment and supportive care. **Table S4.3.** Oseltamivir treatment and chemoprophylactic doses for children and infants.

## Data Availability

The dataset supporting the conclusions of this article are available in “influenza” repository on Github, [DOI: https://github.com/samlipworth/influenza].

## Abbreviations

AGREE: Appraisal of Guidelines for Research and Evaluation II
CDC: Centre for Disease Control and Prevention
CMG: Clinical Management Guidelines
EVD: Ebola virus disease
GPPI: Grupo Promotor de Políticas Informadas
GRADE: The Grading of recommendations, assessment, development and evaluation
GTEI: Grupo de Estudio de Infecciones en el Paciente Crítico
GT-PBE: Grupo de Trabajo de Pediatría Basada en la Evidencia
HCID: High-consequence infectious diseases
HFNC: High-flow nasal cannula
HIC: High-income country
ISARIC: International Severe Acute Respiratory and Emergency Infection Consortium
JMARD: Japan Agency for Medical Research and Development
LMIC: Low- and middle-income countries
MoH: Ministry of Health
NHFPC: China National Health and Family Planning Commission
NIV: Non-invasive ventilation
PAHO: Pan-American Health Organisation
PRISMA: Preferred Reporting Items for Systematic Reviews and Meta-Analyses
PROSPERO: International prospective register of systematic reviews
RCT: Randomised control trials
SR: Systematic review
TRIP: Turning Research Into Practice
UMIC: Upper-middle-income country
US: United States of America
WHO: World Health Organization
