# Supplementary material for: Preparing for pandemics: a systematic review of pandemic influenza clinical management guidelines
Source: BMC Med. 2022 Nov 7;20:425. doi: 10.1186/s12916-022-02616-6 (PMC9640791; doi:10.1186/s12916-022-02616-6)
Supplement: Supplementary file 1 — Additional file 1: Details of the search strategy. S1.1. Database search strategy. S1.1.2. Updated database search strategy. S1.2. Google scholar search strategy. S1.2.2. Updated google scholar search strategy. S1.3. Google engine search strategy. [file 12916_2022_2616_MOESM1_ESM.docx]

# Additional file 1: Search strategy

## S1.1: Database search strategy

Search strategies run on 18^th^ December 2020

| **Databases and guideline repository** | **Strategy** | **Results** |
| --- | --- | --- |
| **Medline (Ovid)** | 1 exp Influenza, Human/co, di, dt, ph, pc, th [Complications, Diagnosis, Drug Therapy, Physiology, Prevention & Control, Therapy] (29817) 2 influenza.ti,kw,fs. (73185) 3 ("H1N1" or "H5N1" or "H7N3" or "H7N7" or "H7N9" or "H9N2").ti,kw,fs. (14611) 4 1 or 2 or 3 (82064) 5 (guideline* or guidance or recommendation* or strategy or "clinical manage*" or standard*).ti,kw,fs. (972368) 6 4 and 5 (2555) 7 (guideline or practice guideline).pt. and humans.sh. (32273) 8 6 and 7 (138) 9 8 (138) 10 limit 9 to yr="2008 -Current" (76) | 76 |
| **Embase**  **(Ovid)** | 1 exp influenza/co, di, dt, pc, th [Complication, Diagnosis, Drug Therapy, Prevention, Therapy] (37347) 2 influenza.ti,kw,fs. (79698) 3 ("H1N1" or "H5N1" or "H7N3" or "H7N7" or "H7N9" or "H9N2").ti,kw,fs. (18555) 4 1 or 2 or 3 (93997) 5 (guideline* or guidance or recommendation* or strategy or "clinical manage*" or standard*).ti,kw,fs. (433183) 6 4 and 5 (1760) 7 exp practice guideline/ (574759) 8 exp human/ (21766586) 9 7 and 8 (508811) 10 6 and 9 (526) 11 10 (526) 12 limit 11 to yr="2008 -Current" (354) | 354 |
| **TRIP** | In the search box: influenza or H1N1 or H5N1 or H7N3 or H7N7 or H7N9 or H9N2 https://www.tripdatabase.com/search?sort=y&criteria=influenza%20or%20H1N1%20or%20H5N1%20or%20H7N3%20or%20H7N7%20or%20H7N9%20or%20H9N2&categoryid=16%2C18%2C10%2C9%2C4 | 0 |
| **Guideline Central** | In the search box: influenza https://www.guidelinecentral.com/?s=influenza%20&dataSource[]=summary&guid=  In the search box: H1N1 https://www.guidelinecentral.com/?s=h1n1  In the search box: H5N1 (no results) https://www.guidelinecentral.com/?s=H5N1  In the search box: H7N3 (no results) https://www.guidelinecentral.com/?s=H7N3 In the search box: H7N7 (no results) https://www.guidelinecentral.com/?s=H7N7 In the search box: H7N9 (no results) https://www.guidelinecentral.com/?s=H7N9 In the search box: H9N2 (no results) https://www.guidelinecentral.com/?s=H9N2 | 0 |
|  | **Total** | 430 |
|  | **Total after deduplication** | 381 |

S1.1.2: Updated database search strategy

Updated on 23^rd^ June 2022

| **Databases and guideline repository** | **Updated Search Strategy** | **Results** |
| --- | --- | --- |
| **Medline (Ovid)** | 1 exp Influenza, Human/co, di, dt, ph, pc, th [Complications, Diagnosis, Drug Therapy, Physiology, Prevention & Control, Therapy]  2 influenza.ti,kw,fs.  3 ("H1N1" or "H5N1" or "H7N3" or "H7N7" or "H7N9" or "H9N2").ti,kw,fs.  4 1 or 2 or 3  5 (guideline* or guidance or recommendation* or strategy or "clinical manage*" or standard*).ti,kw,fs.  6 4 and 5  7 (guideline or practice guideline).pt. and humans.sh.  8 6 and 7  9 8  10 limit 9 to yr="2008 -Current" | 78 |
| **Embase**  **(Ovid)** | 1 exp influenza/co, di, dt, pc, th [Complication, Diagnosis, Drug Therapy, Prevention, Therapy]  2 influenza.ti,kw,fs.  3 ("H1N1" or "H5N1" or "H7N3" or "H7N7" or "H7N9" or "H9N2").ti,kw,fs.  4 1 or 2 or 3  5 (guideline* or guidance or recommendation* or strategy or "clinical manage*" or standard*).ti,kw,fs.  6 4 and 5  7 exp practice guideline/  8 exp human/  9 7 and 8  10 6 and 9  11 10  12 limit 11 to yr="2008 -Current" | 368 |
| **TRIP** | In the search box: influenza or H1N1 or H5N1 or H7N3 or H7N7 or H7N9 or H9N2 https://www.tripdatabase.com/search?sort=y&criteria=influenza%20or%20H1N1%20or%20H5N1%20or%20H7N3%20or%20H7N7%20or%20H7N9%20or%20H9N2&categoryid=16%2C18%2C10%2C9%2C4 | 0 |
| **Guideline Central** | In the search box: influenza https://www.guidelinecentral.com/?s=influenza%20&dataSource[]=summary&guid=  In the search box: H1N1 https://www.guidelinecentral.com/?s=h1n1  In the search box: H5N1 (no results) https://www.guidelinecentral.com/?s=H5N1  In the search box: H7N3 (no results) https://www.guidelinecentral.com/?s=H7N3 In the search box: H7N7 (no results) https://www.guidelinecentral.com/?s=H7N7 In the search box: H7N9 (no results) https://www.guidelinecentral.com/?s=H7N9 In the search box: H9N2 (no results) https://www.guidelinecentral.com/?s=H9N2 | 0 |
|  | **Total** | 446 |
|  |  |  |

## S1.2. Google Scholar search strategy

The Google Scholar search was conducted on the 7^th^ January 2021 and updated on the 16 June 2022. The year was limited from 2008 forward.

| **Search** | **Search terms** | **Results** |
| --- | --- | --- |
| 1 | allintitle: pandemic influenza guideline OR recommendation OR guidance OR consensus OR standard OR management OR summary -cell -lymphocyte -crisis | 235 |
| 2 | allintitle: novel influenza guideline OR recommendation OR guidance OR consensus OR standard OR management OR summary -cell -lymphocyte -crisis | 64 |
| 3 | allintitle: H1N1 guideline OR recommendation OR guidance OR consensus OR standard OR management OR summary -cell -lymphocyte -crisis | 381 |
| 4 | allintitle: H5N1 guideline OR recommendation OR guidance OR consensus OR standard OR management OR summary -cell -lymphocyte -crisis | 53 |
| 5 | allintitle: H7N3 guideline OR recommendation OR guidance OR consensus OR standard OR management OR summary -cell -lymphocyte -crisis | 0 |
| 6 | allintitle: H7N9 guideline OR recommendation OR guidance OR consensus OR standard OR management OR summary -cell -lymphocyte -crisis | 31 |
| 7 | allintitle: H9N2 guideline OR recommendation OR guidance OR consensus OR standard OR management OR summary -cell -lymphocyte -crisis | 1 |
|  | Total | 765 |
|  | Total after deduplication | 635 |

## S1.2.2. Updated google scholar search strategy

Updated on 16 June 2022.

| **Updated Search** | **Search terms** | **Results (2021-2022)** |
| --- | --- | --- |
| 1 | allintitle: pandemic influenza guideline OR recommendation OR guidance OR consensus OR standard OR management OR summary -cell -lymphocyte -crisis | 9 |
| 2 | allintitle: novel influenza guideline OR recommendation OR guidance OR consensus OR standard OR management OR summary -cell -lymphocyte -crisis | 0 |
| 3 | allintitle: H1N1 guideline OR recommendation OR guidance OR consensus OR standard OR management OR summary -cell -lymphocyte -crisis | 4 |
| 4 | allintitle: H5N1 guideline OR recommendation OR guidance OR consensus OR standard OR management OR summary -cell -lymphocyte -crisis | 0 |
| 5 | allintitle: H7N3 guideline OR recommendation OR guidance OR consensus OR standard OR management OR summary -cell -lymphocyte -crisis | 0 |
| 6 | allintitle: H7N9 guideline OR recommendation OR guidance OR consensus OR standard OR management OR summary -cell -lymphocyte -crisis | 0 |
| 7 | allintitle: H9N2 guideline OR recommendation OR guidance OR consensus OR standard OR management OR summary -cell -lymphocyte -crisis | 0 |
|  | Total | 13 |
|  |  |  |

## S1.3. Google engine search strategy

The Google was search was completed on 24^th^ March 2021 and updated on 16^th^ June 2022. The search was conducted in English, Spanish, French Mandarin, Japanese, Italian, Korean, Vietnamese, Hebrew, Dzongkha, Azerbaijani, and Serbian.

| **Search strategy** |
| --- |
| 1. Influenza A virus subtypes* + Clinical management guideline /treatment/guideline 2. Pandemic Influenza + Clinical management guideline /treatment/guideline 3. Influenza + Clinical management guideline /treatment/guideline |
|  |
|  |

***List of Influenza subtypes searched in this study**

H1N1

H5N1

H7N3

H7N7

H7N9

H9N2
