# Supplementary material for: Preparing for pandemics: a systematic review of pandemic influenza clinical management guidelines
Source: BMC Med. 2022 Nov 7;20:425. doi: 10.1186/s12916-022-02616-6 (PMC9640791; doi:10.1186/s12916-022-02616-6)
Supplement: Supplementary file 2 — Additional file 2: Data extraction form. S2.1. Data extraction form. [file 12916_2022_2616_MOESM2_ESM.docx]

# Additional file 2: Data extraction

## S2.1 Data extraction form

| **Assessment group** | **Questions** | **Answer format type** |
| --- | --- | --- |
| **Availability** | Guideline Title | Short-answer text |
|  | Authors | Short-answer text |
|  | Issuing Organisation | Short-answer text |
|  | Date of latest revision | Day, Month, Year |
|  | Is the guideline adapted from another source (please state source) | Short-answer text |
|  | Update process information provided | Yes/No answer |
|  | Publication type (e.g website, journal article, etc) | Short-answer text |
|  | Type of geography | Multiple Choice (Worldwide, Regional, specific to a continent, country specific) |
|  | Geographical aim (e.g., China, UK, USA) | Short-answer text |
|  | Influenza type covered | Short-answer text |
|  | What is the setting? (e.g Low resource setting) | Long-answer text |
| **Inclusivity** | Populations covered | Multiple Choice (Children, Pregnant Women, HIV/Immunocompromised, Older people, Adults) |
|  | Does the guideline mention at-risk groups? | Multiple choice (HIV/immunocompromised, Pregnant Women, Infants Older people, Children, Not specified) |
|  | Age range of older people given? (If yes, please list below) | Short-answer text |
|  | Age range of children given? (If yes, please list below) | Short-answer text |
|  | Age range of infants given? (If yes, please list below) | Short-answer text |
|  | Any other details on population? (if clear- copy & paste) | Long-answer text |
| **Scope** | Is a case definition provided? | Multiple Choice (Yes, No) |
|  | Does the case definition vary by age group? | Yes/No |
|  | What is the Case Definition? (copy & paste if there is a clear one) | Long-answer text |
| **Therapeutic/ Supportive care** | Are antivirals recommended? | Multiple Choice (Yes, no recommendation, yes, conditional, recommended against use) |
|  | Is Amantadine recommended? |  |
|  | Is Rimantadine recommended? |  |
|  | Is Zanamivir recommended? |  |
|  | Is Oseltamivir recommended? |  |
|  | Any other antivirals recommended? |  |
|  | Is Chemoprophylaxis recommended? |  |
|  | Is antiviral resistance Considered? | Yes/No |
|  | Describe the antiviral recommendation for adults (include drugs, doses & age range) - If clear, copy & paste | Long-answer text |
|  | Describe the antiviral recommendation for older people (include drugs, doses & age range) - If clear, copy & paste | Long-answer text |
|  | Describe the antiviral recommendation for children (include drugs, doses & age range) - If clear, copy & paste | Long-answer text |
|  | Describe the antiviral recommendation for infants (include drugs, doses & age range) - If clear, copy & paste | Long-answer text |
|  | Describe the antiviral recommendation during pregnancy (include drugs, doses & age range) - If clear, copy & paste | Long-answer text |
|  | Describe the antiviral recommendation for people living with HIV (include drugs, doses & age range) - If clear, copy & paste | Long-answer text |
|  | Describe the antiviral recommendation by severity (include drugs, doses & age range)- If clear, copy & paste | Long-answer text |
|  | What is the supporting evidence for antiviral recommendations? | Multiple choice (Links to scientific literature, expert consensus, links to another guideline, not specified) |
|  | Anything else interesting regarding antivirals? | Long-answer text |
|  | Are antibiotics recommended? | Multiple Choice (Yes, no recommendation, yes, conditional, recommended against use) |
|  | Which specific antibiotics are recommended? (If clear, copy & paste) | Long-answer text |
|  | Describe the antibiotic recommendations (If clear, copy & paste) | Long-answer text |
|  | What is the supporting evidence for the antibiotic recommendation? | Multiple choice (Links to scientific literature, expert consensus, links to another guideline, not specified) |
|  | Are corticosteroids recommended? | Multiple Choice (Yes, no recommendation, yes, conditional, recommended against use) |
|  | Describe corticosteroid recommendation (If clear, copy & paste) | Long-answer text |
|  | What is the supporting evidence for corticosteroid recommendations? | Multiple choice (Links to scientific literature, expert consensus, links to another guideline, not specified) |
|  | Is fluid therapy recommended? | Multiple Choice (Yes, no recommendation, yes, conditional, recommended against use) |
|  | Describe the fluid therapy recommendations (If clear, copy & paste) | Long-answer text |
|  | Do fluid therapy recommendations differ by population? (If yes- how?) - (If clear, copy & paste) | Long-answer text |
|  | What is the supporting evidence for fluid therapy? | Multiple choice (Links to scientific literature, expert consensus, links to another guideline, not specified) |
|  | Is oxygen therapy discussed? |  |
|  | Is NIV (Non-invasive ventilation) recommended? | Multiple Choice (Yes, no recommendation, yes, conditional, recommended against use) |
|  | Is HFNC (High Flow Nasal Cannula) recommended? |  |
|  | Are target oxygenation levels mentioned? | Yes, No |
|  | Do oxygen therapy recommendations differ by population? (If yes, how?) | Long-answer text |
|  | Describe the oxygen recommendations (If clear, copy & paste) | Long-answer text |
|  | What is the supporting evidence for oxygen therapy? | Multiple choice (Links to scientific literature, expert consensus, links to another guideline, not specified) |
|  | Are any other treatments recommended? (If yes- which?) | Long-answer text |
|  | What is the supporting evidence for any other recommendations? | Multiple choice (Links to scientific literature, expert consensus, links to another guideline, not specified) |
|  | Are there any recommendations for at-home care/management for patients with Influenza? | Yes, No |
|  | Please give more details | Long-answer text |
|  | Are there criteria for discharging patients? | Yes, No |
|  | please give more details | Long-answer text |
|  | Other comments | Long-answer text |
