# Supplementary material for: Preparing for pandemics: a systematic review of pandemic influenza clinical management guidelines
Source: BMC Med. 2022 Nov 7;20:425. doi: 10.1186/s12916-022-02616-6 (PMC9640791; doi:10.1186/s12916-022-02616-6)
Supplement: Supplementary file 3 — Additional file 3: Additional figure Figure S3.1. PRISMA diagram [file 12916_2022_2616_MOESM3_ESM.docx]

# Additional file 3: Additional Figures

## Figure S3.1 PRISMA diagram
