# Supplementary material for: Preparing for pandemics: a systematic review of pandemic influenza clinical management guidelines
Source: BMC Med. 2022 Nov 7;20:425. doi: 10.1186/s12916-022-02616-6 (PMC9640791; doi:10.1186/s12916-022-02616-6)
Supplement: Supplementary file 4 — Additional file 4: Additional tables Table S4.1. Characteristics of identified CMGs. Table S4.2. CMG recommendations for treatment and supportive care. Table S4.3. Oseltamivir treatment and chemoprophylactic doses for children and infants. [file 12916_2022_2616_MOESM4_ESM.docx]

# Additional file 4: Additional Tables

## Table S4.1: Characteristic of identified CMGs

| **CMG** | **HCID** | **Year** | **Issuing Organisation** | **Country/Region** | **Income level*** | **Population covered** | **Quality**** |
| --- | --- | --- | --- | --- | --- | --- | --- |
| Ang, B. et al., [49] | A (H1N1) | 2009 | National | Singapore | HIC | I, C, A, P | 0 |
| Arbo, A^.^ [76] | A (H1N1) | 2009 | National | Paraguay | MIC | I, C, A, O, P, H/Imm, | 0 |
| Aristizábal, G. et al.[51] | A (H1N1) | 2009 | National | Colombia | MIC | I, C, A, | 1 |
| Capozzi, C. et al.[52] | A (H1N1) | 2009 | National | Italy | HIC | Generic+ | 0 |
| Cheng, A. C. et al.[53] | A (H1N1) | 2009 | National | Australasia | HIC | C, A, P, | 0 |
| Cojocaru, V. et al.[54] | A (H1N1) | 2009 | National | Moldova | MIC | I, C, A, P, | 0 |
| Salud Madrid[55] | A (H1N1) | 2009 | National | Spain | HIC | I, C, | 0 |
| Fernández-Cruz, E. et al.[56] | A (H1N1) | 2009 | National | Spain | HIC | C, A, P, H/Imm, | 0 |
| GTEI[57] | A (H1N1) | 2009 | National | Spain | HIC | I, C, A, P, H/Imm, | 2 |
| GPPI[58] | A (H1N1) | 2009 | National | Argentina | MIC | A, P | 0 |
| Hajjar, L. A. et al.[59] | A (H1N1) | 2009 | National | Brazil | MIC | I, C, A, | 0 |
| Mexico[60] | A (H1N1) | 2009 | National | Mexico | MIC | I, C, A, P, O, | 2 |
| El Salvador[61] | A (H1N1) | 2009 | National | El Salvador | MIC | C, A | 0 |
| India[62] | A (H1N1) | 2009 | National | India | MIC | I, C, A, | 0 |
| MOH Argentina[63] | A (H1N1) | 2009 | National | Argentina | MIC | I, C, A, P, O, H/Imm, | 0 |
| MOH Brazil[64] | A (H1N1) | 2009 | National | Brazil | MIC | C, A, P, O | 0 |
| MOH El Salvador[65] | A (H1N1) | 2009 | National | El Salvador | MIC | I, C, A, P, O, H/Imm, | 0 |
| MOH Italy[66] | Pandemic Influenza | 2009 | National | Italy | HIC | C, A, P, | 0 |
| PAHO[92] | A (H1N1) | 2009 | International | The Americas | All | I, C, A, P, O, H/Imm, | 0 |
| PAHO (b)[67] | A (H1N1) | 2009 | International | The Americas | All | C, A, | 1 |
| Picone O. et al.[68] | A (H1N1) | 2009 | National | France | HIC | A, P | 0 |
| Soria, J. et al.[69] | A (H1N1) | 2009 | National | Peru | MIC | I, C, A, P, O H/Imm, | 1 |
| France[70] | A (H1N1) | 2009 | National | France | HIC | I, A, P, | 1 |
| WHO[71] | A (H1N1) | 2009 | International | Global | All | I, C, A, | 1 |
| GT-PBE[72] | A (H1N1) | 2010 | National | Spain | HIC | I, C, | 2 |
| Lee, PI., et al.[73] | A (H1N1) | 2010 | National | Taiwan | MIC | I, C, | 0 |
| MOH Mexico[74] | A (H1N1) | 2010 | National | Mexico | MIC | I, C, A, P, O, H/Imm, | 0 |
| Schaberg, T. et al.[75] | A (H1N1) | 2010 | National | Germany | HIC | Generic+ | 0 |
| Arbo Sosa, A and Araya S.[76] | A (H1N1) | 2011 | National | Paraguay | MIC | A, H/Imm | 0 |
| Fiore, A. E. et al.[77] | Pandemic Influenza | 2011 | National | United States | HIC | I, C, A, P, O, H/Imm, | 1 |
| Hajjar, Al. S. et al.[78] | A (H1N1) | 2011 | International | Eastern Mediterranean | All | C, A, | 0 |
| Zhong, N. et al.[95] | Pandemic Influenza | 2011 | National | China | MIC | C, A, | 0 |
| Rodriguez, A. et al.[79] | A (H1N1) | 2012 | National | Spain | HIC | Generic+ | 1 |
| Evans, A G. et al.[80] | A (H7N9) | 2013 | National | Canada | HIC | I, C, A, | 0 |
| MOH Russia[81] | Pandemic Influenza | 2013 | National | Russia | MIC | C, A, | 0 |
| Saldías, F.[82] | Pandemic Influenza | 2013 | National | Chile | HIC | I, C, A, P, O, H/Imm, | 0 |
| Garcia, C. et al.[83] | Pandemic Influenza | 2014 | National | Chile | HIC | I, C, A, P, | 2 |
| Bin, C. et al.[84] | Pandemic Influenza | 2016 | National | China | MIC | I, C, A, P, O | 0 |
| US CDC[96] | Pandemic Influenza | 2016 | National | United States | HIC | Generic+ | 0 |
| British Columbia[85] | Pandemic Influenza | 2017 | National | Canada | HIC | I, C, A, | 0 |
| China NHFPC[93] | A (H7N9) | 2017 | National | China | MIC | C, A, | 0 |
| JAMRD[86] | Pandemic Influenza | 2017 | National | Japan | HIC | Generic+ | 0 |
| MOH Colombia[87] | Pandemic Influenza | 2018 | National | Colombia | MIC | I, C, A, P, | 0 |
| Taiwan CDC[88] | Influenza A | 2018 | National | Taiwan | MIC | Generic+ | 0 |
| China NHFPC[89] | Pandemic Influenza | 2020 | National | China | MIC | I, C, A, | 0 |
| Fernandez, O. et al.[90] | Pandemic Influenza | 2020 | National | Bolivia | MIC | I, C, A, P, O, H/Imm | 0 |
| WHO[94] | Pandemic Influenza | 2022 | International | Global | All | I, C, A, P, O, H/Imm, | 2 |
| MOH Japan[91] | A (H5N1) | - | National | Japan | HIC | Generic+ | 0 |
| **Abbreviations:** HIC: High income country; MIC: Middle income country; U: Unspecified: P:Pregnant women; C:Children; H/Imm: HIV/Immunocompromised; O: Older people; I: Infants,  HCID: High consequence infectious disease, CMG: Clinical management guidelines, MoH: Ministry of Health, CDC: Center for disease control and prevention, JAMRD: Japan Agency for Medical Research and Development, NHFPC: National Health and Family Planning Commission, WHO: World Health Organisation, PAHO: Pan-American Health Organisation, GT-PBE: Grupo de Trabajo de Pediatría Basada en la Evidencia | | | | | | | |
| *Income level based on World Bank  + Generic guidelines did not clearly state the populations covered | |  |  |  |  |  |  |
|  | | | |  |  |  |  |

**Quality as per AGREE II tool: Green=high quality (≥ 60% in domain three and two other non-specified domains),

Yellow =medium quality (≥ 60% in any three or more non-specified domains but not including domain three),

Orange=low quality (less than three domains scored ≥60%)

## Table S4.2: CMG Recommendations for treatment and supportive care

|  |  |  |  | **Supportive Care** | | | | | | **Treatment** | | | | | |
| --- | --- | --- | --- | --- | --- | --- | --- | --- | --- | --- | --- | --- | --- | --- | --- |
| **CMG** | **HCID** | **Year** | **Country/ Region** | **Oxygen** | **Fluid** | **NIV** | **HFNC** | **At-home care** | **Discharge criteria** | **Oseltamivir** | **Zanamivir** | **Rimantadine** | **Amantadine** | **Cortico-steroids** | **Antibiotics** |
| Ang, B. et al., [49] | A (H1N1) | 2009 | Singapore | - | - | - | - | - | - | Y | Y | Y | Y | - | - |
| Arbo, A^.^ [76] | A (H1N1) | 2009 | Paraguay | Y | Y | Y* | Y* | Y | Y | Y* | Y* | - | - | - | Y* |
| Aristizábal, G. et al.[51] | A (H1N1) | 2009 | Colombia | Y | Y* | Y* | Y* | Y | Y | Y | - | - | - | - | Y* |
| Capozzi, C. et al.[52] | A (H1N1) | 2009 | Italy | Y* | Y* | - | - | - | - | Y | Y | CI | CI | - | - |
| Cheng, A. C. et al.[53] | A (H1N1) | 2009 | Australasia | - | - | - | - | - | - | Y* | Y* | - | - | - | CI |
| Cojocaru, V. et al.[54] | A (H1N1) | 2009 | Moldova | Y | Y* | - | - | - | - | Y* | - | - | - | Y* | Y* |
| Salud Madrid[55] | A (H1N1) | 2009 | Spain | - | - | - | - | Y | - | Y* | Y* | CI | CI | - | - |
| Fernández-Cruz, E. et al.[56] | A (H1N1) | 2009 | Spain | - | - | - | - | Y | - | Y | Y* | - | - | - | Y* |
| GTEI[57] | A (H1N1) | 2009 | Spain | - | - | - | - | Y | - | Y | Y* | CI | CI | - | - |
| GPPI[58] | A (H1N1) | 2009 | Argentina | - | - | - | - | - | - | Y | Y | - | - | - | - |
| Hajjar, L. A. et al.[59] | A (H1N1) | 2009 | Brazil | Y* | Y* | Y* | - | - | - | Y | Y | - | - | Y* | Y* |
| Mexico[60] | A (H1N1) | 2009 | Mexico | - | - | - | - | Y | - | Y* | Y* | - | CI | - | Y* |
| El Salvador[61] | A (H1N1) | 2009 | El Salvador | Y | Y* | - | - | Y | Y | Y* | Y* | CI | CI | - | Y* |
| India[62] | A (H1N1) | 2009 | India | Y | Y | Y* | Y* | Y | Y | Y | - | - | - | Y* | Y* |
| MOH Argentina[63] | A (H1N1) | 2009 | Argentina | - | - | CI | - | - | Y | Y* | Y* | - | - | - | - |
| MOH Brazil[64] | A (H1N1) | 2009 | Brazil | - | - | - | - | Y | - | Y* | Y* | - | - | - | - |
| MOH El Salvador[65] | A (H1N1) | 2009 | El Salvador | Y | Y | Y* | Y* | Y | - | Y* | Y* | - | - | - | Y* |
| MOH Italy[66] | Pandemic  Influenza | 2009 | Italy | - | - | - | - | - | - | Y* | Y* | - | - | - | - |
| PAHO[92] | A (H1N1) | 2009 | The Americas | Y | Y | Y* | Y* | Y | - | Y* | Y* | - | - | Y* | Y* |
| PAHO (b)[67] | A (H1N1) | 2009 | The Americas | Y | Y | - | - | Y | Y | Y | Y | - | - | - | - |
| Picone O. et al.[68] | A (H1N1) | 2009 | France | Y | N | - | - | - | - | Y | Y | - | - | - | Y* |
| Soria, J. et al.[69] | A (H1N1) | 2009 | Peru | Y | N | CI | - | Y | - | Y* | Y* | - | - | - | Y* |
| France[70] | A (H1N1) | 2009 | France | Y* | N | Y* | - | Y | - | Y* | Y* | - | - | - | - |
| WHO[71] | A (H1N1) | 2009 | Global | Y* | N | - | Y* | - | - | Y | Y | - | - | Y* | Y* |
| GT-PBE[72] | A (H1N1) | 2010 | Spain | - | - | - | - | Y | - | Y* | Y* | CI | CI | - | Y* |
| Lee, PI., et al.[73] | A (H1N1) | 2010 | Taiwan | Y | N | CI | - | - | - | Y | Y | - | - | CI | Y* |
| MOH Mexico[74] | A (H1N1) | 2010 | Mexico | Y* | N | Y* | Y* | Y | Y | Y* | - | - | - | - | Y* |
| Schaberg, T. et al.[75] | A (H1N1) | 2010 | Germany | Y | N | Y | - | - | - | Y | Y | - | - | - | - |
| Arbo Sosa, A and Araya S.[76] | A (H1N1) | 2011 | Paraguay | - | - | - | - | Y | - | Y* | Y* | CI | CI | - | - |
| Fiore, A. E. et al.[77] | Pandemic  Influenza | 2011 | United States | - | - | - | - | - | - | Y | Y | CI | CI | - | Y* |
| Hajjar, Al. S. et al.[78] | A (H1N1) | 2011 | Eastern Mediterranean | - | - | - | - | Y | Y | Y* | Y* | - | - | Y* | Y* |
| Zhong, N. et al.[95] | Pandemic  Influenza | 2011 | China | Y | Y* | Y* | Y* | Y | - | Y* | Y* | Y* | Y* | Y* | - |
| Rodriguez, A. et al.[79] | A (H1N1) | 2012 | Spain | Y* | N | CI | Y* | - | - | Y | Y | CI | CI | CI | Y* |
| Evans, A G. et al.[80] | A (H7N9) | 2013 | Canada | Y | N | - | - | - | - | Y* | Y* | - | - | CI | - |
| MOH Russia[81] | Pandemic  Influenza | 2013 | Russia | Y | N | - | - | Y | Y | Y* | Y* | CI | - | Y | Y* |
| Saldías, F.[82] | Pandemic  Influenza | 2013 | Chile | - | Y* | - | - | Y | - | Y | Y* | CI | CI | - | - |
| Garcia, C. et al.[83] | Pandemic  Influenza | 2014 | Chile | - | - | - | - | Y | - | Y | Y | CI | CI | - | - |
| Bin, C. et al.[84] | Pandemic  Influenza | 2016 | China | - | - | - | - | - | - | Y | Y | CI | CI | Y* | - |
| US CDC[96] | Pandemic  Influenza | 2016 | United States | Y | N | Y* | Y* | Y | - | Y* | Y* | CI | CI | - | Y* |
| British Columbia[85] | Pandemic  Influenza | 2017 | Canada | - | - | - | - | Y | - | Y* | - | - | - | - | - |
| China NHFPC[93] | A (H7N9) | 2017 | China | - | - | - | - | - | - | Y* | Y* | CI | CI | Y* | Y* |
| JAMRD[86] | Pandemic  Influenza | 2017 | Japan | Y | N | CI | Y* | - | - | Y* | Y* | - | - | Y* | - |
| MOH Colombia[87] | Pandemic  Influenza | 2018 | Colombia | Y | Y | Y* | Y* | Y | - | Y* | - | - | - | - | Y* |
| Taiwan CDC[88] | Influenza A | 2018 | Taiwan | Y* | N | - | - | - | - | Y | Y | CI | CI | Y* | Y* |
| China NHFPC[89] | Pandemic  Influenza | 2020 | China | Y* | N | Y* | Y* | Y | - | Y | Y | CI | CI | Y* | Y* |
| Fernandez, O. et al.[90] | Pandemic  Influenza | 2020 | Bolivia | - | - | - | - | - | - | Y* | Y* | - | - | - | - |
| WHO[94] | Pandemic Influenza | 2022 | Global | - | - | - | - | - | - | Y * | CI | - | - | CI | CI |
| MOH Japan[91] | A (H5N1) | - | Japan | Y* | N | - | - | - | Y* | Y* | - | - | CI | Y* | Y* |
| **Abbreviations**: CI: contraindicated; NIV: non-invasive ventilation; HFNC: high flow nasal cannula, MoH: Ministry of Health, CDC: Center for disease control and prevention, JAMRD: Japan Agency for Medical Research and Development, NHFPC: National Health and Family Planning Commission, WHO: World Health Organisation, PAHO: Pan-American Health Organisation, GT-PBE: Grupo de Trabajo de Pediatría Basada en la Evidencia | | | | | | | | | | | | | | | |

* conditions provided for the use of these interventions (including severity, suspected complications, fever, etc.)

## Table S4.3: Oseltamivir treatment and chemoprophylactic doses for children and infants

| Children and Infants | |
| --- | --- |
| Oseltamivir Treatment | Oseltamivir Chemoprophylactic |
| Duration: 5 days  If ≥ 1 years old,  ≤ 15 kg: 30 mg twice per day/ 60 mg once per day 15–23 kg: 45 mg twice per day/ 90 mg once per day 24–40 kg: 60 mg twice per day/ 120 mg once per day > 40 kg 75 mg twice per day/150 mg once per day  If 3 – < 12 months: 3 mg/kg/dose twice per day  If 0 – < 3 months: 3 mg/kg/dose twice per day | Duration: 10 days  ≤ 15 kg: 30 mg twice per day/ 60 mg once per day  15–23 kg: 45 mg twice per day/ 90 mg once per day  24–40 kg: 60 mg twice per day/ 120 mg once per day  > 40 kg 75 mg twice per day/150 mg once per day  3 – < 12 months:  3 mg/kg/dose twice per day  0 – < 3 months: 3 mg/kg/dose twice per day |
